# Unilateral or bilateral V-Y fasciocutaneous flaps for the coverage of soft tissue defects following total knee arthroplasty

**DOI:** 10.1186/1749-799X-5-82

**Published:** 2010-11-04

**Authors:** Konstantinos Papaioannou, Stergios Lallos, Andreas Mavrogenis, Elias Vasiliadis, Olga Savvidou, Nikolaos Efstathopoulos

**Affiliations:** 1 2nd Academic Department of Trauma and Orthopaedics, School of Medicine, Kapodistrian University, Athens, Greece; 2 Orthopaedic Department, "Thriasio" General Hospital, G. Gennimata Av. 19600, Magoula, Athens, Greece; 3 Plastic and Reconstructive Department, Oncology IKA Hospital "G.Gennimatas", str. Asopiou 4, Athens, Greece

## Abstract

**Background:**

Soft tissue necrosis following total knee arthroplasty (TKA) may be the cause of the devastating complication of deep infection. It necessitates an immediate operative intervention because it could potentially jeopardise the arthroplasty or even the limb.

**Methods:**

Sixteen consecutive patients with a mean age of 73,8 years (range 47 to76 years) over a 6-year period (January 2003 to December 2008) with wound dehiscence after TKA were enrolled in the present study. Unilateral or bilateral fasciocutaneous V-Y flaps that are differently oriented, depending on the local conditions of the tissues were used to reconstruct the soft tissues defects.

**Results:**

In 15 of the 16 cases studied, the wound was successfully covered with the presented technique while in 1 patient a partial flap loss occurred, which was healed after surgical debridement and the application of vacuum system. No other complications occurred. Knee prosthesis was salvaged in all the patients with a good functional and esthetical outcome.

**Conclusions:**

The presented reconstructive technique is a simple, quick, versatile and reliable solution for the coverage of soft tissue defects following TKA, more than 2 cm width and grade 1 and 2 according to Laing classification, provided the V-Y flaps are applied early in the postoperative period and no complex defects are involved.

## Background

The potentially disastrous complication of an infection after total knee arthroplasty (TKA) often is heralded by the delay of wound healing or soft tissue necrosis, and may jeopardize the prosthesis. The exposed knee prosthesis poses a challenge to the orthopaedic surgeon. The incidence of severe wound problems after TKA that is, those requiring a second return to the operating room ranges from 0,33% to 5,3% [1]. Wound problems could be a superficial skin loss or more severe necrosis of large areas of skin and subcutaneous tissues with implant exposure, which may go on to deep infection of the prosthesis [2-5]. Some form of immediate operative intervention may then be indicated [6].

Several predisposing factors such as immuno-suppression, malnutrition, diabetes mellitus, steroid use, rheumatoid arthritis, previous incisions, smoking, obesity and vascular disease can be involved in the onset of wound complications, as well as long tourniquet time and aggressive early postoperative knee flexion [7-10].

Knee prostheses are particularly at risk because of their relative superficial location. Even though there is no consensus in the management of soft tissue defects following TKA, well-planned strategies are necessary for sufficient soft tissue reconstruction, including local wound care, debridement, and soft tissue coverage with skin or muscle flaps, resulting in optimal functional and aesthetic results. The inadequate coverage after arthroplaty also has the disadvantage of preventing early motion of the knee joint. Non-operative management, especially when the soft tissue defect persists for several weeks, may fail because the thin subcutaneous tissues about the knee already provide minimum coverage of the underlying prosthesis. Local cutaneous flaps may also be ineffective, while inclusion of the deep fascia with the panniculus adiposus affords a safer transposition [11].

This study presents the unilateral or bilateral V-Y fasciocutaneous flaps technique for the coverage of soft tissue defects more than 2 cm width following TKA and emphasises the need for early plastic surgery consultation. Although the V-Y fasciocutaneous flap is a well-known technique for coverage of soft tissue defects [24] to our knowledge there are no published studies concerning the defects following TKA. Indications and restrictions of the specific technique are also discussed.

## Methods

**Figure 1:**
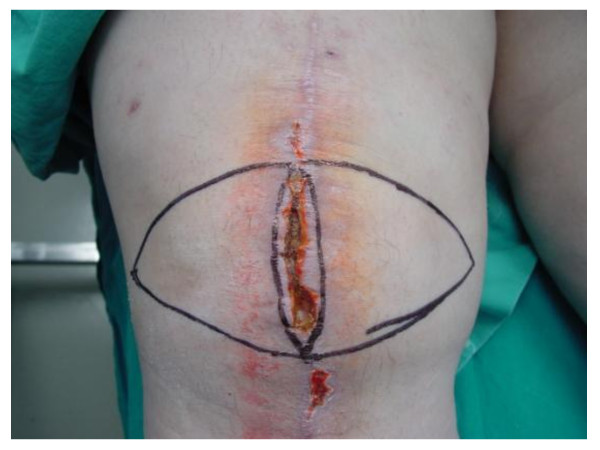
**Wound dehiscence following TKA in an obese patients**. Skin dehiscence that is apparent was the top of the "ice-berg" with a larger amount of necrosed tissue underneath the skin and subsequent larger amount of tissue to be removed with debridement. Preoperative drawing of unilateral V-Y fasciocutaneous local flap.

**Figure 2:**
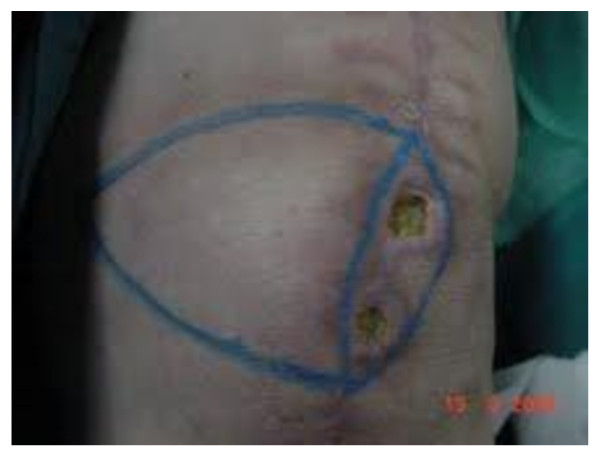
**Wound dehiscence following TKA**. Preoperative drawing of bilateral V-Y fasciocutaneous local flap.

A total of sixteen consecutive patients (6, 2%) over a 6-year period (January 2003 to December 2008) with wound dehiscence following TKA were enrolled. Fifteen were female and one male. All had undergone TKA for primary knee osteoarthritis. The mean age of patients was 73,8 years (range 47 to76 years). The mean time of wound breakdown since TKA was 35 days (range 14 to 56 days). The severity of wound dehiscence was classified according to the Laing classification [12] (Table 1). Systemic antibiotics were administered to all patients on the basis of wound swab culture results, which were taken prior to any procedure (Table 2). Soft tissue cover was achieved using either a unilateral or bilateral V-Y fasciocutaneous local flaps with different orientation (Figures 1, 2). The mean time of follow up was 28 months (range 6 to 60 months).

**Table 1:** The severity of wound dehiscence according to the Laing classification

Grade	Extend of wound dehiscence
0	Simple erythema, no superficial necrosis
1	Skin necrosis and wound breakdown, no sinus into the joint
2	Extensive skin necrosis with a wound sinus into the joint
3	Deep wound dehiscence with a sinus, little or no prosthetic exposure
4	Deep wound dehiscence, with overt prosthetic exposure

**Table 2:** Microbiological wound swab cultures

Patients	Age	Sex	Overweight	Diabetes	Other Diseases	Wound cultures
1	70	F	Yes	No	HT	negative
2	74	F	Yes	No		negative
3	76	F	Yes	No		negative
4	74	F	Yes	Yes	HT, RA	negative
5	76	F	Yes	Yes	HT	negative
6	47	F	Yes	No		negative
7	73	F	Yes	No	HT	negative
8	69	F	Yes	No	HT	Staphylococcus Aureus
9	69	F	Yes	No		Staphylococcus Heamolyticus, Enterococcus Faecium, Pseudomonas Aeruginosa
10	74	F	Yes	No	Heavy Smoker	Staphylococcus Aureus, Enterococcus Avium and Faecium, Corynobacterium Striatum
11	53	M	No	No	HT, RA	Staphylococcus Epidermidis
12	63	F	Yes	Yes		Candida
13	73	F	Yes	Yes	HT, Smoker	Enterococcus Faecium
14	71	F	Yes	No		Negative
15	75	F	Yes	No		Negative
16	70	F	Yes	No		Negative

HT, Hypertension; RA, Reumatoid Arthritis

### Surgical Technique

Initially the wound is debrided and incorporated in the medial aspect of an assumed fusiform excision, horizontally or oblique oriented. The height and width of each separated triangle depends on the dimensions of the deficit to be covered plus the stiffness and oedema of the local tissues. The incision is carried down to the fascia. On the medial aspect, flap elevation may necessitate to sacrifice the greater saphenous nerve and vein. Care must be taken when elevating the medial flap at the attachment of the tendons of the sartorius, gracilis, and semitendinous muscles. This medial flap is based on the saphenous artery and vein. If a similar flap is elevated laterally, care should be taken to avoid injury to the common peroneal nerve, which is superficial at the proximal fibula. No suction drain is needed.

Postoperatively, the patient is bed resting with splinting of the involved knee for 2 weeks. Following the period of bed rest, partial weight bearing is recommended. Knee flexion begins at 3 weeks postoperatively.

## Results

**Figure 3:**
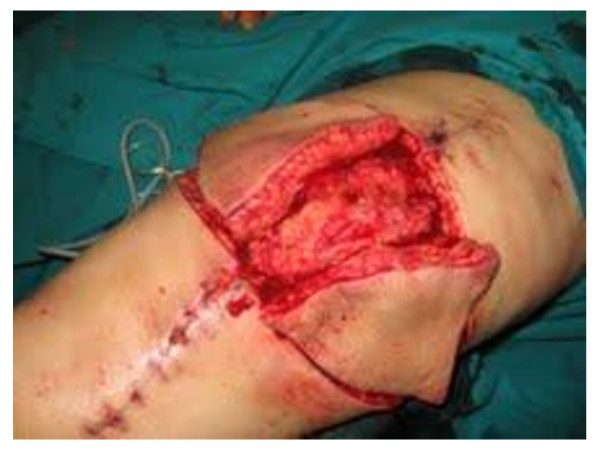
**Bilateral V-Y flap: the incision is carried down to the fascia**.

**Figure 4:**
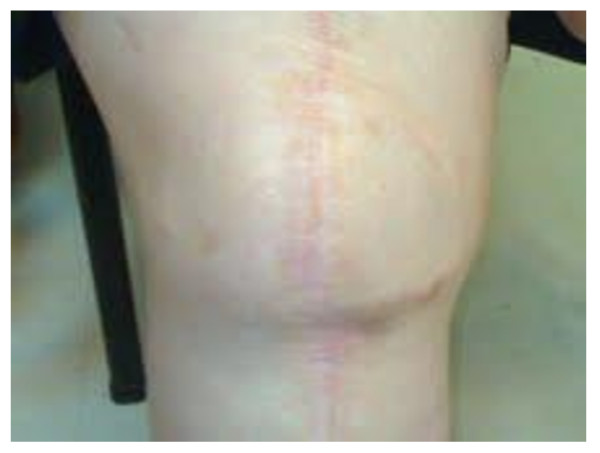
**Final result 12 months post-operatively**.

The dimensions of the deficits ranged from 2 × 5 cm to 4,5 × 12 cm. According to the Laing grading system 15 of the patients had grade 1 wound dehiscence and 1 had grade 2. Wound swab cultures were positive in 6 patients, but none of them had the arthrosis or the implant infected. Thirteen patients were treated by bilateral flaps (Figure 3) while in 3 patients a unilateral flap was adequate. All patients achieved a good final outcome, with good range of motion of the knee joint at the latest follow-up. None of the 16 patients mentioned any sensory deficit (numbness) round the knee area after the V-Y flap. Fifteen wounds healed without any complication (Figure 4). Only one patient with grade 1 skin necrosis had a partial flap loss unilaterally at its central and peripheral part, probably due to a poor local circulation affected by the diabetes and a heavy panniculus pad. This partial flap loss was healed conservatively after surgical debridement and the use of vacuum system, with no need of prosthesis replacement. No other complications occurred.

## Discussion

Poor wound healing after TKA can lead to devastating complications. The risk seems to be major in the presence of factors that affect local vascularity to the soft tissues [8,13]. Skin vascularity over the knee affects the rate of healing postoperatively and the risk of necrosis. Since the beginning of TKA in 1971, most surgeons recommend a straight anterior midline approach for TKA in patients without previous scars of the knee [14]. Anastomoses of the femoral and popliteal arteries supply blood to the skin on the anterior knee. Although the skin blood feeding depends heavily on the terminal branches of the anterior anastomoses, there is a better blood supply originating medially [15,16]. Ries measured transcutaneous skin oxygen tension and found that the oxygen tension decreases for the first 2 to 3 days after surgery, then increases again [17]. In addition, the lateral skin edge is more hypoxic than the medial edge. This implies that more medial-based incisions tend to interrupt dermal blood supply closer to its source, leaving the lateral incision edge compromised. A more laterally based incision would theoretically leave intact a skin perfusion that originates medially. A recent study depicts lymphatic drainage of the leg originating from the foot, crossing over to the medial side of the knee at or just opposite to the tibial tubercle, suggesting that incisions are not to be placed too medially [16].

The pattern of blood supply throughout the lower extremity is longitudinally oriented. Through numerous anastomoses, an axial direction of cutaneous blood flow is enhanced, which provides the basis for safety in raising long and narrow local fasciocutaneous flaps around the knee. The flap should be based along the axially oriented pattern of vascularisation to ensure the integrity of the circulation when the fasciocutaneous flap is raised.

Repairing of a soft tissue defect after TKA is usually not a simple surgical procedure, as the direct suturing is ineffective most of the times. If the prosthesis is not exposed and the defect is small fasciocutaneous flaps may be more suitable for coverage than are flaps that sacrifice muscles function [11,18]. Defects of more than 2 cm width (including debridement tissues at the margins of the wound) is an indication for unilateral or bilateral V-Y flaps without any tension at the central suturing line. A V-Y flap is an advancement flap that leaves the tissue to slide toward the defect for a distance almost equal to the height of the Y. That gives the advantage of adequate movement of the flaps without any tension at the periphery of the flap and the skin edges [24,25]. In certain areas such as the frontal area of the knee where other types of skin or fasciocutaneous flaps are inadequate in terms of designing and arc of rotation, the advancement of the V-Y flaps in an horizontal manner parallels the relaxed tension lines leaving a very satisfactory functional and esthetic result. If bone or tendons are exposed, especially when the prosthesis is uncovered, a musculocutaneous flap (medial or lateral gastrocnemius) or even free flaps are the methods of choice [19,20]. Muscle flap surgery is considered for grade 3 and 4 wound dehiscence according to Laing grading system [2]. Misra et al [21] found the fascial feeder - and perforator- based local fasciocutaneous flap in the patellar and peripatellar regions to be a reproducible technique to perform. By islanding local flaps on perforator/fascial feeder vessels, greater mobility is achievable, when compared to conventional flaps. Combining local fascial feeder-and perforator-based flaps with V-Y advancement minimizes donor site complication. Lately the pedicled descending genicular artery (DGAP) arises from the medial side of the superficial femoral artery approximately 13 cm above the medial joint line of the knee. This flap can be used as a free tissue transfer because of its long vascular leash (up to 15 cm), its relatively large arterial calibre (1.5 to 2 mm), its rapid and straightforward dissection for flap elevation and its thin and minimally hirsute skin and anatomically distinct nerve supply that allows provision of sensate flaps. However, universal acceptance of the flap has been limited due to the variations of the vascular anatomy that make the planning and elevation of this flap somewhat more challenging than other similar options [22]. Nevertheless, the elevation of fasciocutaneous flaps single or double in a V-Y manner for the coverage of less extensive defects requires less tissue sacrifice and leaves the underlying muscles intact, reserving them for future use as an alternative surgical procedure. In addition, the application of a fasciocutaneous flap in an infected trauma due to its adequate vascularity is considered superior to an "ischemic" skin flap. However, if the arthrosis and the implant are infected then the use of a pedicled or free muscle flap is preferred.

Whenever flap surgery is not the treatment of choice in treating difficult wound defects due to the high risk of failure, negative pressure plays a significant role. However it necessitates a long period of hospital stay with a lot of dressings and bed immobilization that may prolong the period of knee immobilization and probably affect the functional results [23]. For these reasons it was not the first choice of treatment and used in only one case after the partial flap loss with satisfactory final results.

If poor wound healing or skin necrosis occurs after TKA, early recognition of the problem minimizes the risk of deeper infection and necrosis. There is no agreement about the stage that intervention should occur, but adequate wound care, including detection of infection, debridement, and early appropriate defect coverage, should be the main points to consider. Early awareness of the surgeons should prevent more complex tissue necrosis with or without involvement of the prosthesis. Consider that fat necrosis of subcutaneous tissues, if any, appears by the 15^th ^to 21^st ^day postoperatively and that necrotic eschar has to be clearly defined, the best period for the reoperation is between 3-4 weeks after initial operation. However if the procedure is applied later it is not a contraindication, provided that the necrosis is not ongoing and the joint stiffness is not as such severe as it may affect the final range of knee motion.

Regarding the rehabilitation programme, it is inevitable that if soft tissue necrosis appears after TKA the rehabilitation of the patient is delayed. The earlier (according to the indications) this surgical technique is performed, the better for the rehabilitation schedule of the patient. Mobilization of the knee joint in this group of 16 patients started at 2 to 3 weeks postoperatively, and all the patients achieved good range of knee motion. As long as this technique is usually uneventful and reserves all other reconstructing techniques with muscle flaps or free flaps for more complicated cases, final mobilization of the patient is considered early compared with conservative regime or direct re-suturing (with the risk of a new necrosis) that may delay more the rehabilitation and even decrease the range of knee motion.

## Conclusions

The V-Y fasciocutaneous flaps reconstructive technique is a versatile and reliable solution for the coverage of soft tissue defects following total knee arthroplasty, grade 1 and 2 according to Laing classification and more than 2 cm width (including debridement tissues at the margins of the wound) provided the V-Y flaps are applied early in the postoperative period and no complex defects are involved. It provides a series of advantages such as, a simple and quick surgical procedure, a well vascularized tissue bulk which is enhanced by the delay phenomenon due to the previous surgical approach, a usually uneventful postoperative period, a quicker mobilisation of the patient, the reservation of other reconstructive alternatives in case of any serious further complication, a minimum compromise of an already disabled extremity and a satisfactory functional and cosmetic result.

## Competing interests

The authors declare that they have no competing interests.

## Authors' contributions

KP and NE conceived the idea of the presented study, performed part of the literature review and contributed in drafting of the manuscript and in the interpretation of data. SL, AM, EV and OS performed part of the literature review and contributed in the manuscript editing. All authors have read and approved the final manuscript.
